# Effects of Perfluorooctanoic Acid on the Associated Genes Expression of Autophagy Signaling Pathway of *Carassius auratus* Lymphocytes *in vitro*

**DOI:** 10.3389/fphys.2018.01748

**Published:** 2018-12-05

**Authors:** Juan Tang, Xiangjun Lu, Feifei Chen, Xueping Ye, Dongren Zhou, Julin Yuan, Jianbo He, Bin Chen, Xiaodong Shan, Jinxiao Jiang, Wenli Liu, Hangjun Zhang

**Affiliations:** ^1^College of Life and Environmental Sciences, Hangzhou Normal University, Hangzhou, China; ^2^Zhejiang Institute of Freshwater Fisheries, Huzhou, China; ^3^Key Laboratory of Hangzhou City for Ecosystem Protection and Restoration, Hangzhou Normal University, Hangzhou, China

**Keywords:** autophagy, oxidative stress, perfluorooctanoic acid, *Carassius auratus*, lymphocyte

## Abstract

Perfluorooctanoic acid (PFOA) has been detected in various water bodies and caused harm to aquatic organisms. The aim of this study was to investigate the cytotoxicity and mechanism associated with autophagy and oxidative stress after exposure to PFOA (0, 1, 10, 100 μg/L) for 12 h on lymphocytes, which was isolated from the head kidney of *Carassius auratus* (*C. auratus*). Both of autophagy formation, cell activity, and intracellular reactive oxygen species (ROS), malondialdehyde (MDA), glutathione (GSH), and superoxide dismutase (SOD) levels were measured. The relative expression of partial autophagy-related genes autophagy related 5 (Atg 5), autophagy related 7 (Atg 7), and Beclin 1 were also cloned and detected. Homologous relationships analysis showed high identities of genes in *C. auratus* and other fish by blast. *C. auratus* lymphocytes growth inhibition rates was increased induced by PFOA. Compared with the control group, the ROS generation and the MDA content were significantly increased in all of the PFOA-treated group. Besides, decreased SOD activity and decrease of GSH activity induced by PFOA further confirmed the occurrence of oxidative stress. The number of autophagosome formations was increased in a dose-dependent manner. Compared with the control group, Atg 7 and Beclin 1 mRNA expression was elevated significantly after PFOA exposed, showing a time-dependent manner, while mRNA expression of Atg 5 was increased remarkably in 100 μg/L PFOA-treated group. Our results indicated that PFOA caused oxidative damage to lymphocytes in *C. auratus* and caused various autophagy signaling pathway-associated genes imbalances in the lymphocytes. Autophagy signaling pathway-associated genes imbalance could weaken antioxidant capacity and involve in the mechanism of *C. auratus* lymphocytes oxidative injury caused by PFOA.

## Introduction

Perfluorinated compounds (PFCs), with their unique chemical and thermal stabilities, have been widely used for over 50 years in various industries and consumer product (Giesy and Kannan, 2001; Han and Fang, 2010). The physico-chemical characteristics of PFCs cause extreme difficulties due to their degradation, thereby converting them into hazardous pollutants that accumulate in biota and water on a global scale (Prevedouros et al., 2006), and perfluorooctanoic acid (PFOA) is one type of PFCs. PFOA is easily detected in water bodies because of its excellent water solubility and refractory character, especially in parts of China (Niu et al., 2016). The concentrations of PFOA were 46.88 and 58–1594.83 ng/L in the estuaries of the Yangtze River and the Huangpu River, respectively (Zhang et al., 2006). PFOA is easily detected in water and has a significant impact on aquaculture due to its excellent water solubility and refractory character. PFOA accumulates in the muscle, liver, kidneys, gills, blood, skin, and carcass of fish by the mouth and gills, and then leads to various toxicities (Giari et al., 2016). The kidneys are important immune organs for fish, PFOA accumulation in the kidney is the main mechanism of immunotoxicity in fish and the elimination of PFOA by trout occurs primarily via the renal route (Consoer et al., 2014).

Over recent decades, various potential bioeffects of PFOA have been demonstrated using *in vivo* and *in vitro* models (Upham et al., 2009). PFOA has significant biological toxicity, and its immunotoxicity has become the main research subject. PFOA concentrations observed in serum are associated with lower antibody responses to childhood immunizations such as diphtheria and tetanus, as well as childhood deficits in immune system functions (Midgett et al., 2015). PFOA can affect human immune cells mainly through natural killer-cell cytotoxicity and pro-inflammatory cytokine release by stimulated macrophages (Brieger et al., 2011). PFOA exposure has been shown to decrease T-cell-dependent IgM antibody responses in mice (DeWitt et al., 2009). Most studies regarding the immunotoxicity of PFOA were performed in humans and mice, but studies regarding the immunotoxicology in aquatic organisms remain unclear. PFOA can generally cause oxidative stress by producing reactive oxygen species (ROS) and lipid peroxidation in aquatic organisms and mammals both *in vivo* or *vitro* (Liu et al., 2007; Eriksen et al., 2010; Zhao et al., 2011; Hagenaars et al., 2013). A report indicated that Perfluorooctane sulfonate exposure induced autophagy blockade in the liver, an immune organ (Yao et al., 2014). Additionally, the results provided a new direction for the immunotoxicity mechanism induced by PFOA and whether it will cause lymphocytes injury *in vitro* has not been reported so far.

Autophagy is an intracellular degradation system and a non-injurious response to maintain cellular homeostasis and constitutes stress adaptation that prevents cell death in the case of changing internal and external conditions in eukaryotes (Mizushima, 2007). A review showed that autophagy performed multitiered immunological functions that influence immunity (Deretic et al., 2013). Autophagy protein 5 is encoded by the Atg5 gene in humans. Both Atg5 and autophagy protein 7 (Atg7) play an important role in the elongation of autophagosomes, while Atg7 activates the cascade to form autophagosomes (Juhász et al., 2007; Miller et al., 2008; McCoy et al., 2010). Beclin 1 also plays an important role in the autophagy of a catabolic process of degradation induced by starvation, being implicated in autophagic programmed cell death (Zhong et al., 2009). Recent research has indicated that ROS were emerging as important players in autophagy (Scherz-Shouval et al., 2007; Scherz-Shouval and Elazar, 2011). Therefore, uncontrolled ROS and defective proteins were removed by mitochondrial autophagy to regulate autophagy process (Lee et al., 2012). Autophagy was demonstrated to remove “older” peroxisomes in rat liver and showed a protective effect of autophagy to oxidative stress induced by PFOA (Cavallini et al., 2017). However, the reports about the effects of Atg5, Atg7, and Beclin 1 on the *Carassius auratus* lymphocytes *in vitro* are rare.

It remains unknown whether PFOA also cause *C. auratus* lymphocytes oxidative damage *in vitro*, and what the mechanism of autophagy and oxidative stress induced by PFOA in aquatic organisms, especially fish. We hypothesized that PFOA could induced oxidative damage to lymphocytes in *C. auratus* and caused various autophagy signaling pathway-associated genes imbalances in the lymphocytes. Thus, the current study evaluated the effect of autophagy in *C. auratus* lymphocytes oxidative damage process exposed to different doses of PFOA. Furthermore, the possible underlying molecular mechanism of *C. auratus* lymphocytes oxidative damage was investigated with a specific focus on autophagy signaling pathway-associated genes, including Atg 5, Atg 7, and Beclin 1. The results of this study may provide a theoretical basis for the protection of the cytotoxicity mechanism of PFOA in aquatic products.

## Materials and Methods

### Reagents

PFOA (C_8_HF_15_O_2_, CAS No. 335-67-1, >96% purity, molecular weight = 414.06) was purchased from Sigma-Aldrich (St. Louis, MO, United States). The experimental concentration gradient was set according to the results of the early stage of our research group for PFOA in aquatic organisms, which was 0, 1, 10, 100 μg/L (Zhang et al., 2014).

### Experimental Fish and Ethic Statement

*Carassius auratus* (*C. auratus*) (6–10 months of age, 400–500 g of weight) were acquired from the hatchery of the Freshwater Fisheries Institute (Zhejiang, China). All *C. auratus* were reared in re-circulating water under environmental conditions (25 ± 2°C), and each *C. auratus* daily had pellet food equal to 0.7% of their body weight. Two weeks later, healthy *C. auratus* were used for this study. This study was carried out in accordance with the principles of the Basel Declaration and recommendations of Hangzhou Normal University Experimental Animal Center. The protocol was approved by the Hangzhou Normal University Experimental Animal Ethics Committee.

### Isolation and Treatment of *C. auratus* Lymphocytes

*Carassius auratus* kidneys removed methods were according to our previous study (Zhang et al., 2013). The kidney tissues were passed through a nylon sieve, and single-cell suspensions were obtained by teasing tissues in serum-free Roswell Park Memorial Institute (RPMI)-1640 culture medium. Next, the cells were layered onto a 1.5-volume lymphoprep system (Qiyuan Biological, Shanghai, China; density adjusted to 1.077 g/mL). *C. auratus* lymphocytes were collected and washed three times with phosphate-buffered saline (PBS) after centrifuging at 650 × *g* for 30 min. The cells were counted by a hemocytometer. 5% fetal calf serum (CO_2_, 27°C) used for cell culture, and which was no antibiotic RPMI-1640 medium (Hangzhou Keyi Shengwu, Hangzhou, China). In a 96-well plate at a density of 1 × 10^6^ cells/well, *C. auratus* lymphocytes were incubated with 0, 1, 10, 100 μg/L PFOA for 12 h. The concentration gradient was set according to the content of PFOA in parts of water bodies in China (Chen et al., 2009).

### Autophagosome Assay

*Carassius auratus* lymphocytes cells were stained and analyzed using a Cyto-ID Green Kit (ENZO, United States) according to the manufacturer’s instruction. Cells were then observed under a laser scanning confocal microscope (LSM 700, ZEISS, Germany).

### 3-(4,5-Dimethythiazol-2-yl)-2,5-Diphenyl Tetrazolium Bromide (MTT) Cytotoxicity Assay

3-(4,5-Dimethythiazol-2-yl)-2,5-Diphenyl Tetrazolium Bromide (MTT) test was used to determine the effect of PFOA on *C. auratus* lymphocytes. MTT kit was purchased from Beyotime Institute of Biotechnology (Shanghai, China) and 5 mg/mL of MTT was used to incubate the cells. Then, removed the medium and dissolved the violet crystals using dimethyl sulfoxide. Finally, the absorbance at 570 nm was measured by a microplate reader.

### ROS Generation Assays

Dichloro-dihydro-fluorescein diacetate (DCFH-DA) was used to detect the ROS generation in *C. auratus* lymphocytes, which was purchased from Beyotime Institute of Biotechnology (Shanghai, China). After incubation with different concentrations of PFOA for 12 h, collected and homogenized the *C. auratus* lymphocytes in cold PBS. Subsequently, cells were treated with a DCFH-DA (10 μM concentration, 20 min, room temperature). Finally, at excitation and emission wavelengths of 488 and 525 nm, Guava EasyCyte 8HT flow cytometer was used to analysis the cells, which was in the InCyte mode.

### MDA Content, GSH and SOD Activities Assay

Use cold PBS to collect and homogenize the *C. auratus* lymphocytes. To obtain the supernatants, homogenates were centrifuged at 650 × *g* for 15 min at 4°C. The glutathione (GSH) and malondialdehyde (MDA) contents of cells were detected using assay kits (Jiancheng, Nanjing, China) according to the manufacturer’s instructions.

Superoxide dismutase (SOD) activities were determined following the procedures of the enzyme activity assay kit (WST-1). The supernatants were treated with the appropriate reagents in the SOD assay kit, and the mixture was incubated at 37°C for 20 min. Using a multimode microplate reader (Infinite M1000), SOD activity at 450 nm was measured according to the manufacturer’s instructions. Assay kits were purchased from Nanjing Jiancheng Bioengineering, Inc. (Jiangsu, China).

### Total RNA Extraction

Total RNA was extracted from the lymphocytes of *C. auratus* using the Trizol reagent (Invitrogen, Carlsbad, CA, United States) according to the manufacturer’s instructions. DNA contamination was removed by RNase-free DNase 1 (Takara, Dalian, China) treatment. The quantity of total RNA was estimated by agarose gel electrophoresis and UV spectrophotometry (Implen GmbH, Munich, Germany).

### Molecular Cloning of Partial Atg 5, Atg 7, and Beclin 1

Total RNA used to obtain partial Atg 5, Atg 7, and Beclin1 cDNA was extracted from the lymphocytes of *C. auratus*. Total RNA from each sample was reverse transcribed to cDNA with an Omniscript R Reverse Transcription kit (Takara) with Oligo-dT primers (Takara) according to the manufacturer’s instructions and used for RT-PCR. 1 μg total RNA was reverse transcribed to first-strand cDNA by SuperScript^TM^III First-strand Synthesis SuperMix (Invitrogen, United States). Analysis the three genes’ cDNA to design the primers and amino acid sequences of various fish species that have been reported. Degenerate primers were designed according to the conservation of their sequences. Beacon Designer 7.8 software and Primer Premier 6.0 were used to confirm the core fragments and ensure sequence accuracy. Then the synthesis was completed by Bioethics Engineering (Shanghai, China). The primer sequences were shown in Table 1. Agarose gel electrophoresis used to separate the appropriate size amplified PCR products. A Zymoclean Gel DNA Recovery kit (Zymo Research, Orange, CA, United States) used to purify and cloned into a pGM-T vector (Takara, Dalian, China), detected. Then sequenced by Bioethics Engineering (Shanghai, China). The PCR amplification reaction system and conditions are shown in Table 2.

**Table 1:** Primer sequences used for detecting of gene relative expression by real-time PCR in this study (Primer sequences of ATG5, ATG7, and Beclin1 were designed based on the cDNA sequences and the ORF fragment found by blastx).

Gene	GenBank accession number	Primer sequences (5′–3′)
ATG5	MF 285268(cloned)	F: 5′-CCCTACTATCTGCTCCTCCTACG-3′
		R: 5′-GCGTTCCCTCATATTCTAACCACATC-3′
ATG7	MF 285269(cloned)	F: 5′-CAGTCCCTCATTTTCTGCTCAAGTA-3′
		R: 5′-GGTCTGGGAAGAAGGAGGTCAAGTC-3′
Beclin1	MF 285270(cloned)	F: 5′-GTGTCTCGGAAATACATCCCAC-3′
		R: 5′-GTCACTTTCAGCCTGCGACTC-3′
β-Actin	AB039726.2	F: 5′-′CACTTCCCTTGCTCCTTCCAC-3′
		R: 5′-GAAGGGCCAGACTCATCGTACT-3′

**Table 2:** Nucleotide sequence homology of Atg 5, Atg 7, and Beclin 1 between *Carassius auratu*s and other fish.

Atg 5	*Carassius auratus*
*Sinocyclocheilus rhinocerous*	98%
*Danio rerio*	97%
*Salmo salar*	96%
**Atg 7**	*Carassius auratus*
*Sinocyclocheilus rhinocerous*	98%
*Sinocyclocheilus anshuiensis*	94%
*Salmo salar*	78%
**Beclin 1**	*Carassius auratus*
*Danio rerio*	95%
*Oryzias latipes*	89%
*Gasterosteus aculeatus*	89%

### Sequence Analysis

Software suite Lasergene v 7.0 (DNASTAR, Madison, WI, United States) was used to assemble and analysis the sequence data. Open reading frames and proteins were predicted according to the NCBI ORF Finder^1^. Homologous nucleotide and protein sequences were confirmed using the blastn and blastx search algorithm in NCBI^2^. Multiple alignments of amino acid sequences were performed using the clustal X^3^. The Atg 5 homolog sequences registered in GenBank shown follows: *Sinocyclocheilus rhinocerous*, Atg 5 (XP_016383287.1); *Danio rerio*, Atg 5 (NP_991181.2); *Salmo salar*, Atg 5 (NP_001167283.1). The Atg 7 homolog sequences registered in GenBank shown follows: *D. rerio*, Atg 7 (XP_016431353.1); *Oryzias latipes*, Atg 7 (XP_016340089.1); *Gasterosteus aculeatus*, Atg 7 (NP_001158792.1). The Beclin1 homolog sequences registered in GenBank are as follows: *D. rerio*, Beclin1 (NP_957166.1); *Oryzias latipes*, Beclin 1 (NP_001098248.1); *Gasterosteus aculeatus*, Beclin1 (NP_001254561.1).

### Quantitative Real-Time PCR

The cDNA templates used for quantitative RT-PCR analysis were generated using the method described above and were further amplified with the β-actin primer (Table 1) to exclude any possible residual DNA contamination. The primer sequences of Atg5, Atg7, and Beclin1 were the same as degenerate primers used in molecular cloning (Table 1). qRT-PCR was performed using a CFX384 Touch^TM^ Real-Time PCR Detection System (Bio-Rad, United States) in 384-well plates (20 μL reaction volume). The relative gene expression levels of each partial gene fragment were measured using a standard curve generated. The threshold cycle Ct was determined for each real-time PCR assay. Each gene’ Ct values were normalized to that Ct values of the housekeeping gene (β-actin). Gene expression changes were quantified using the 2^−ΔΔCT^ method (Yu et al., 2007), and each sample was analyzed six times.

### Statistical Analysis

Data were expressed as mean ± standard deviation (SD) and analyzed by one-way analysis of variance (ANOVA) with the appropriate *post hoc* test (Dunnet’s multiple comparison test). All calculations and statistical analyses were performed with GraphPad Prismsoftware (GraphPad Software, San Diego, CA, United States). Partial correlation analysis was performed on nine parameters. Differences were considered to be statistically significant at a *p*-value < 0.05.

## Results

### Cytotoxicity Induced by PFOA in *C. auratus* Lymphocytes

**FIGURE 1:**
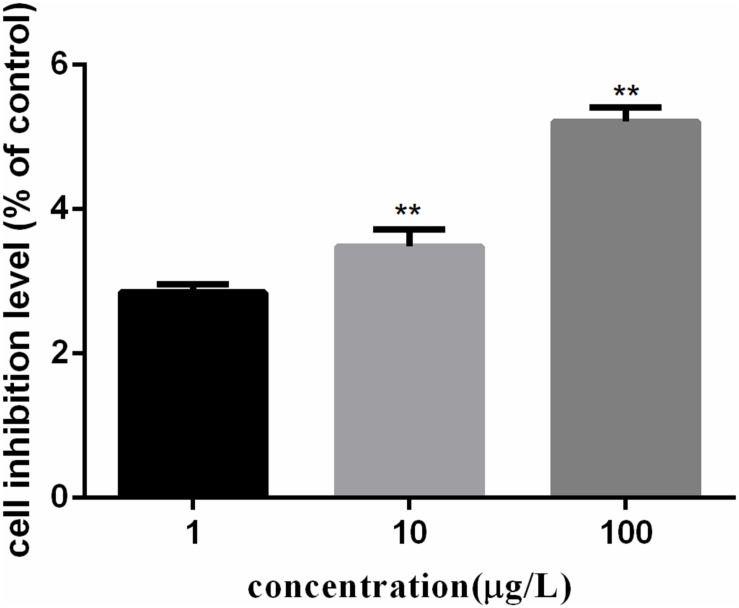
PFOA-induced growth inhibition rates of *Carassius auratus* lymphocytes. Cells treated with different concentrations (1, 10, and 100 μg/L) of PFOA for 12 h. Growth inhibition rates of *C. auratus* lymphocytes treated with PFOA increased significantly compared with those of untreated groups. Data are represented as the mean ± SD. Significant difference from the control was determined by ^∗∗^*P* < 0.01.

The growth inhibition rates of *C. auratus* lymphocytes induced by PFOA were detected by the MTT cell proliferation assay and using a cytotoxicity assay kit. Figure 1 showed the cell growth inhibition rates of five exposed groups compared with untreated groups. The growth inhibition rate of lymphocytes treated with 0.001 mg/L PFOA totaled 2.85% (*P* < 0.01).

### Correlation Between Autophagy and Oxidative Stress in *C. auratus* Lymphocytes

**FIGURE 2:**
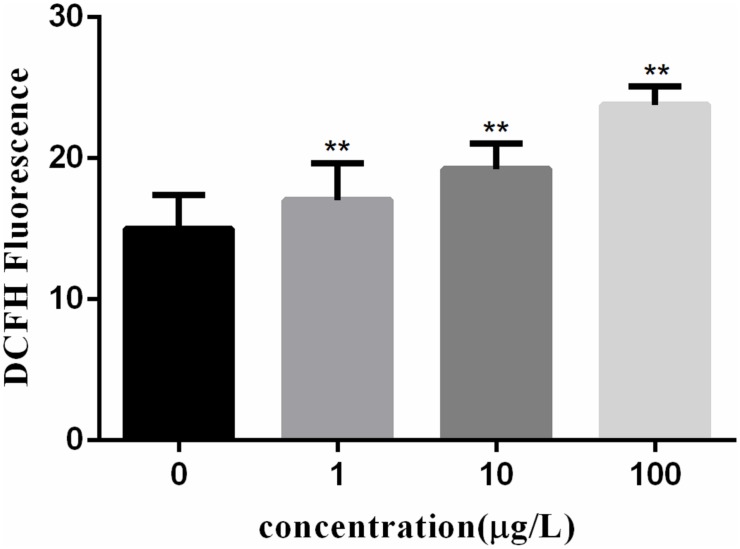
Effect of PFOA on ROS activity in *C. auratus* lymphocytes. Data are represented as the mean ± SD. Significant difference from the control was determined by ^∗^*P* < 0.05 and ^∗∗^*P* < 0.01.

Reactive oxygen species generation in cells was represented by DCF fluorescence in Figure 2. The ROS levels in lymphocytes exposed to PFOA for 12 h were increased significantly in a dose-dependent pattern. When cells were exposed to 1–100 μg/L of PFOA for 12 h, the ROS levels were significantly increased (16.03, 18.24, 22.77%; *P* < 0.01).

**FIGURE 3:**
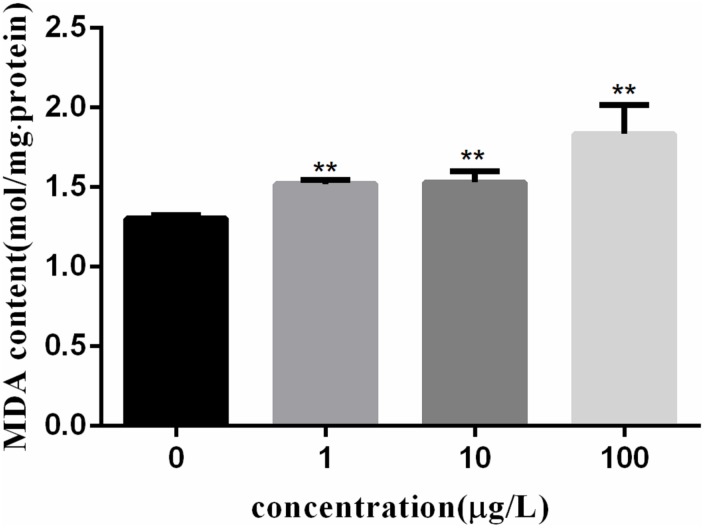
Effect of PFOA on MDA content in *C. auratus* lymphocytes. Data are represented as the mean ± SD. Significant difference from the control was determined by ^∗∗^*P* < 0.01.

Figure 3 shows the MDA content in fish lymphocytes exposed to PFOA for 12 h. The MDA content in lymphocytes was increased by 27.6% (*P* < 0.01) after 1 μg/L of PFOA treatment. The MDA content in cells was increased with increasing PFOA concentration.

Table 3 shows the GSH levels in fish lymphocytes exposed to PFOA. The GSH level in cells treated with PFOA (1–100 μg/L) was decreased remarkably (*P* < 0.01). Table 3 also provides the effect of PFOA on SOD activity in lymphocytes. SOD activity in lymphocytes was significantly decreased with the increase in the exposure concentration of PFOA. When the exposure concentration of PFOA was the lowest (1 μg/L), SOD activity was decreased by approximately 9%. SOD activity was showed significantly decreased (*P* < 0.01) in the PFOA treatment group compared with the control group. These results indicate that SOD activity can be decreased by PFOA *in vitro*.

**Table 3:** Results of GSH content and SOD activity in *Carassius auratus* lymphocytes induced by PFOA.

	**Control**	**PFOA (μg/L)**		
	
	**0**	**1**	**10**	**100**
GSH (mg/L)	6.62 ± 0.07	5.93 ± 0.07^∗∗^	5.51 ± 0.06^∗∗^	4.903 ± 0.06^∗∗^
SOD (U/mgprot)	85.30 ± 0.41	77.84 ± 0.53^∗∗^	71.58 ± 0.56^∗∗^	63.37 ± 0.72^∗∗^

*Values are expressed as mean ± SD (n = 6). ^∗∗^ Significantly different from control (p < 0.01).*

### Molecular Characterization of Atg5, Atg7, and Beclin 1 in *C. auratus* Lymphocytes

**FIGURE 4:**
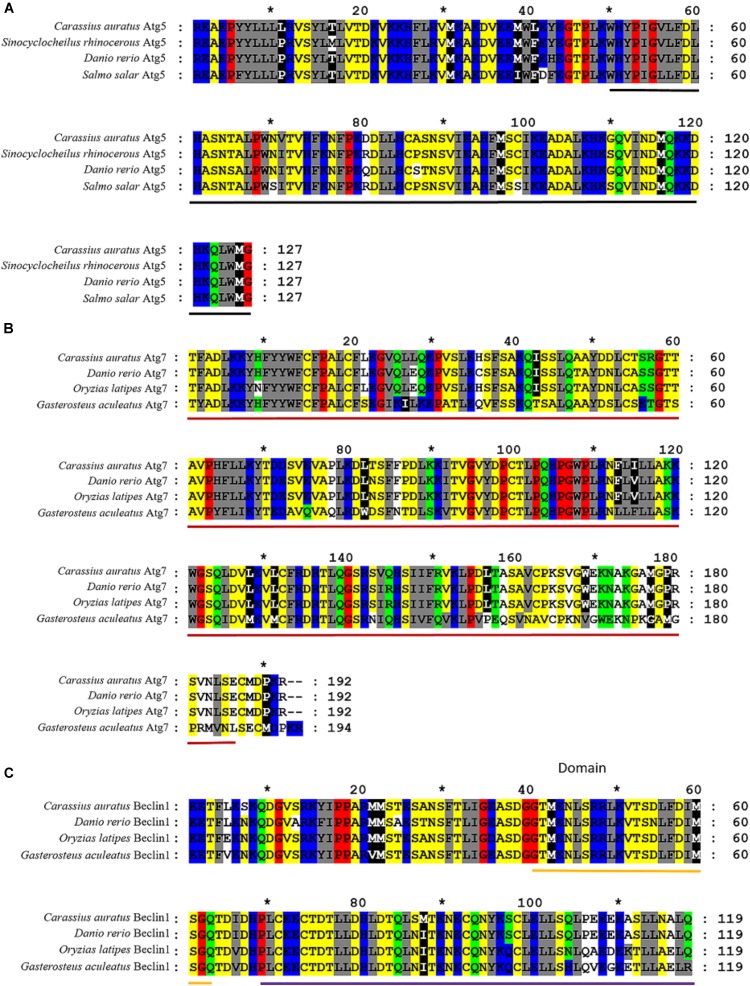
Alignments of amino acid sequences of Atg5, Atg7, and Beclin 1 between *C. auratus* and other fish. **(A)** Alignments of Atg5. Relevant amino acid sequences were obtained from the NCBI GenBank database: *Sinocyclocheilus rhinocerous* (XP_016383287.1), *Danio rerio* (NP_991181.2), and *Salmo salar* (NP_001167283.1). **(B)** Alignments of Atg7. Relevant amino acid sequences were obtained from the NCBI GenBank database: *D. rerio* (NP_957166.1), *Oryzias latipes* (NP_001098248.1), and *Gasterosteus aculeatus* (NP_001254561.1). **(C)** Alignments of Beclin 1. Relevant amino acid sequences were obtained from the NCBI GenBank database: *D. rerio* (XP_016431353.1), *Oryzias latipes* (XP_016340089.1), and *Gasterosteus aculeatus* (NP_001158792.1). Hyphens denote that bases contain gaps with those in the top line. The black bold indicated an autophagy protein 5 domain in Figure 1. The red bold indicated a N-terminal domain of Ubiquitin-like modifier-activating enzyme ATG7 in Figure 1. The yellow and purple bold indicated a BH3 domain and an autophagy protein Apg6 domain in Figure 1, respectively.

Partial cDNA sequences of Atg 5 (382 bp, GenBank Accession No. MF 285268), Atg 7 (577 bp, GenBank Accession No. MF 285269) and Beclin 1 (358 bp, GenBank Accession No. MF 285270) were identified from *C*. *auratus* lymphocytes. The deduced amino acid sequence of Atg 5, Atg 7, and Beclin 1 revealed homology to previously characterized autophagy genes in other fish (Figure 4). Homology analysis by blastn and blastx revealed the homologous relationship of Atg 5, Atg 7, and Beclin 1 between *C. auratus* and other fish species (Table 2). These results suggest the presence of Atg 5, Atg 7, and Beclin 1 transcription factors in *C. auratus* lymphocytes. The amino acid sequences of *C. auratus* Atg 5 showed high identity with *Sinocyclocheilus rhinocerous* Atg 5 (98% homology, 2 different sites of 127 amino acids), *D. rerio* Atg 5 (97% homology, 3 different sites of 127 amino acids), and *Salmo salar* Atg 5 (96% homology, 4 different sites of 127 amino acids). The amino acid sequence identities of Atg 7 between *C. auratus and Sinocyclocheilus rhinocerous*, *Sinocyclocheilus anshuiensis*, and *Salmo salar* reached 98, 94, and 78%, respectively. The amino acid sequence of *C. auratus* Beclin 1 yielded 95, 89, and 89% identity values with *D. rerio, Oryzias latipes*, and *Gasterosteus aculeatus*, respectively.

### PFOA-Induced Autophagy in *C. auratus* Lymphocytes

**FIGURE 5:**
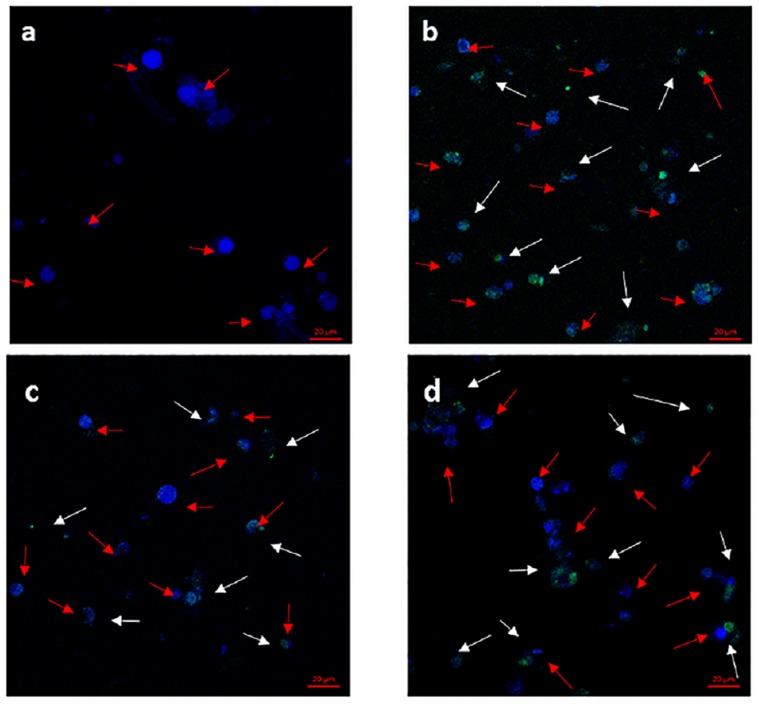
Formation of autophagosomes monitored in *C. auratus* lymphocytes stained with CYTO-ID Green Detection Reagent (green) and counter stained with Hoechst dye (blue) and analyzed by confocal microscopy. Red arrows point to stained nucleus, and white arrows point to green autophagosomes. Cells were exposed to PFOA (1 and 100 μg/L) for 12 h or 60 μM of chloroquine for 6 h. **(a)** Negative control group; **(b)** positive control group; **(c)** *C. auratus* lymphocytes treated with 1 μg/L PFOA, **(d)** *C. auratus* lymphocytes treated with 100 μg/L PFOA.

Confocal microscopy images of lymphocytes exposed to PFOA after 12 h (Figure 5). Laser scanning confocal microscopy demonstrated green fluorescent globular structures, autophagosomes that were formed for the sequestration of intracellular components following exposure, and blue fluorescent globular structures. The red arrows indicate stained nuclei, and white arrows indicate green autophagosomes. Compared to the control group, autophagosomes showed increased in 1 and 100 μg/L PFOA treatment group in *C. auratus* Lymphocytes (Figures 5c,d). Additionally, the number of autophagosomes was increased similar to that of the positive control in the 100 μg/L PFOA treatment group.

**FIGURE 6:**
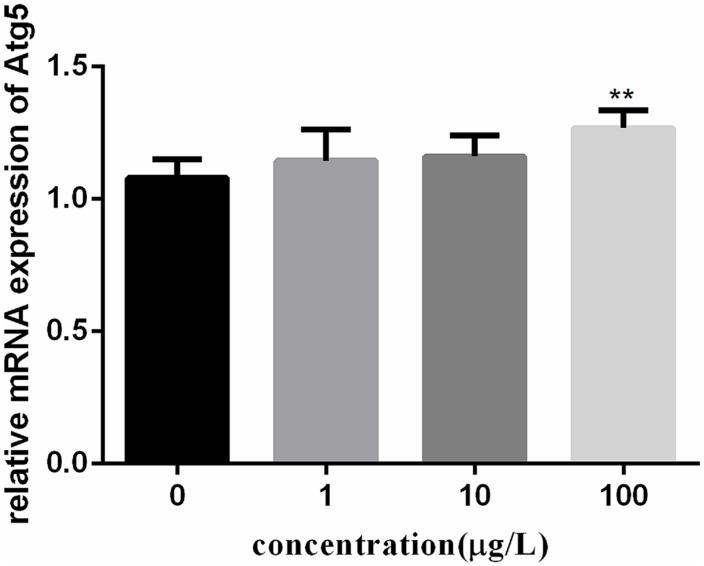
Effect of PFOA on Atg5 mRNA levels in *C. auratus* lymphocytes exposed to 0, 1, 10, and 100 μg/L PFOA for 12 h. Data are represented as the mean ± SD. Significant difference from the control was determined by ^∗∗^*P* < 0.01.

**FIGURE 7:**
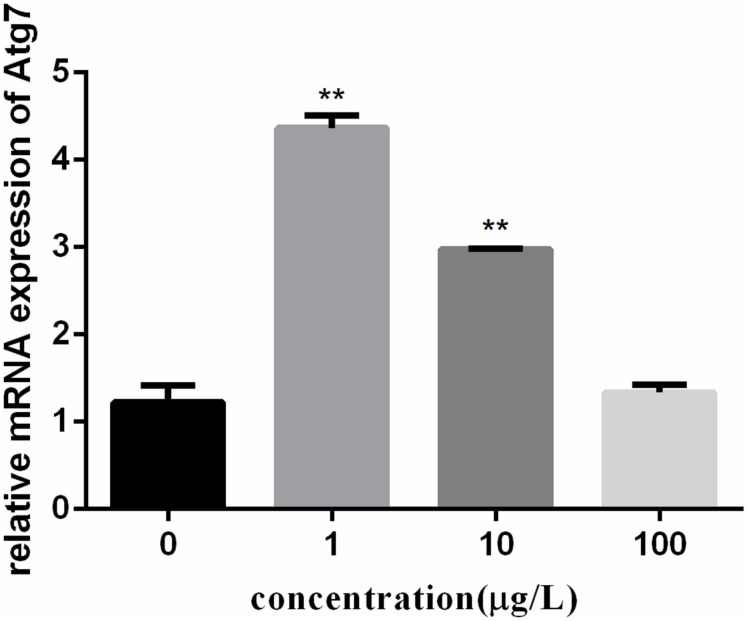
Effect of PFOA on Atg7 mRNA levels in *C. auratus* lymphocytes exposed to 0, 1, 10, and 100 μg/L PFOA for 12 h. Data are represented as the mean ± SD. Significant difference from the control was determined by ^∗∗^*P* < 0.01.

**FIGURE 8:**
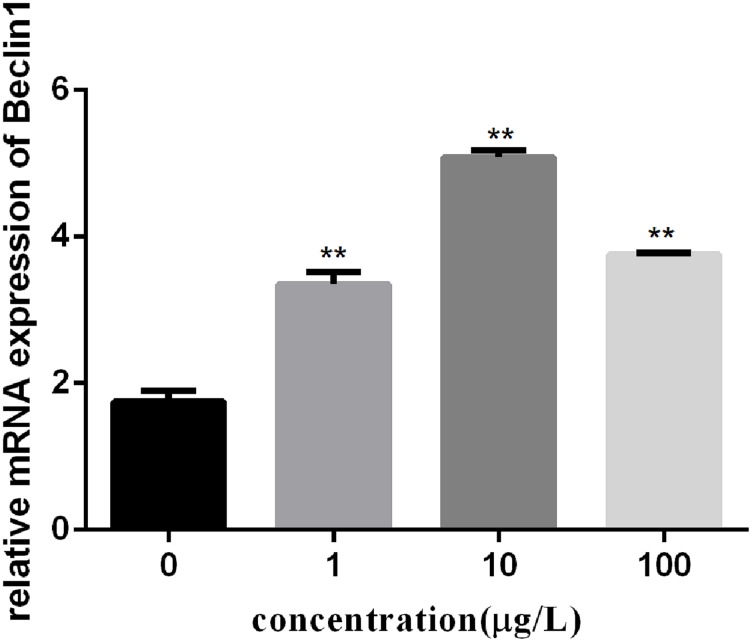
Effect of PFOA on Beclin 1 mRNA levels in *C. auratu* lymphocytes exposed to 0, 1, 10, and 100 μg/L PFOA for 12 h. Data are represented as the mean ± SD. Significant difference from the control was determined by ^∗∗^*P* < 0.01.

To investigate the autophagy mechanism induced by PFOA (12 h) in lymphocytes, the relative expression of a range of important genes in cell autophagy was investigated: Atg 5, Atg 7, and Beclin 1. The data demonstrated the up-regulated expression levels of these genes following exposure to 0, 1, 10, 100 μg/L PFOA (Figures 6–8). However, surprisingly, with the increase in the exposure concentration, the expression of Atg 7 showed a down-regulation trend compared with the 1 μg/L PFOA exposure group, and the relative gene expression of Beclin 1 in the 10 μg/L PFOA exposure group was 1.4-fold higher than that in the 100 μg/LPFOA treatment group. In addition, the correlation coefficient results of the eight parameters were showed in Table 4.

**Table 4:** The correlation coefficient results of the eight parameters.

	MTT	Atg5	Atg7	Beclin1	ROS	MDA	GSH	SOD
MTT	1.00	−0.86^∗∗^	−0.96^∗∗^	−0.86^∗∗^	0.94^∗∗^	0.88^∗∗^	−0.92^∗∗^	−0.92^∗∗^
Atg5	−0.86^∗∗^	1.00	0.70^∗∗^	0.94^∗∗^	−0.97^∗∗^	−0.78^∗∗^	0.79^∗∗^	0.80^∗∗^
Atg7	−0.96^∗∗^	0.70^∗∗^	1.00	0.69^∗∗^	−0.81^∗∗^	−0.81^∗∗^	0.86^∗∗^	0.86^∗∗^
Beclin1	−0.86^∗∗^	0.94^∗∗^	0.69^∗∗^	1.00	−0.93^∗∗^	−0.74^∗∗^	0.76^∗∗^	0.76^∗∗^
ROS	0.94^∗∗^	−0.97^∗∗^	−0.81^∗∗^	−0.93^∗∗^	1.00	0.90^∗∗^	−0.91^∗∗^	−0.92^∗∗^
MDA	0.88^∗∗^	−0.78^∗∗^	−0.81^∗∗^	−0.74^∗∗^	0.90^∗∗^	1.00	−1.00^∗∗^	−1.00^∗∗^
GSH	−0.92^∗∗^	0.79^∗∗^	0.86^∗∗^	0.76^∗∗^	−0.91^∗∗^	−1.00^∗∗^	1.00	1.00^∗∗^
SOD	−0.92^∗∗^	0.80^∗∗^	0.86^∗∗^	0.76^∗∗^	−0.92^∗∗^	−1.00^∗∗^	1.00^∗∗^	1.00

*^∗∗^p < 0.01.*

## Discussion

This study demonstrated that PFOA had an adverse effect on the cytotoxicity, oxidative stress and the Atg 5, Atg 7, and Beclin 1 genes expression in the *C. auratus* lymphocytes. Little is known about the effects of PFOA on the lymphocytes function, and it is the first report about effects of PFOA on the gene expression of Atg 5, Atg 7, and Beclin 1 in the lymphocytes of *C. auratus.* Also we firstly revealed the connection between dysregulation of Atg 5, Atg 7, and Beclin 1 mRNA expression and oxidative damage in *C. auratus* lymphocytes exposed to PFOA.

Liu et al. (2007) demonstrated that PFOA can decrease the viability of hepatocytes of freshwater tilapia in a dose-dependent manner. Hu and Hu (2009) investigated the effects of PFOA on the growth of Hep G2 cells using the MTT method, and the results indicated a dose- and concentration-dependent decrease in cell viability with exposure to PFOA. Multiple studies have shown that ROS are pivotal in PFOA-induced dysfunction (Kleszczynski et al., 2009), and lipid peroxidation is commonly used as a marker of the oxidative stress response in aquatic animals (Lushchak et al., 2001; Dalton et al., 2002). The ROS and MDA contents were increased accompanying an increased exposure concentration of PFOA in this study. Similarly, Wielsøe et al. (2015) reported that PFOA increased ROS generation significantly in a human hepatoma cell line with an exposure time of 24 h. The increased MDA content following PFOA exposure may be attributed to the excessive generation of ROS, enhancing the oxidation of polyunsaturated fatty acids and leading to lipid peroxidation (Liu et al., 2007). Fish, similar to many other vertebrates, attempt to reduce the damage of oxidative stress using antioxidant defense systems. This phenomenon was also demonstrated in a previous study on PFOA in zebrafish liver, where a significant reduction in SOD activity was observed (Liu et al., 2008). In the present study, PFOA can induce cytotoxicity and inhibit the growth of lymphocytes, indicating a dose-dependent effect in low-dose exposure. SOD activity in cells was decreased evidently at the lowest exposure concentration of PFOA. However, several studies presented different results. Yang discovered that PFOA can significantly inhibit CAT at a dose of 50 mg/L with no changes in SOD activity in the liver of male Japanese medaka (*Oryzias latipes*) (Yang, 2010). Cavallini et al. (2017) noted that autophagy may degrade older peroxisomes with the PFOA treatment time during administration in male Sprague–Dawley rats. These results proved that PFOA-induced oxidative damage in the *C. auratus* lymphocytes. In addition, due to previous study has shown that liver injury caused by environmental toxicant exposure can be restored (Tang et al., 2016), we suspect this damage could be recovered after termination of PFOA exposure.

Glutathione is the main redox buffer of the cell and localized in the cytosol (Kern and Kehrer, 2005). SOD converts superoxide into H_2_O_2_, which is further detoxified into H_2_O and O_2_ by catalase or peroxidases (Dorval and Hontela, 2003). Given the role of ROS in inducing autophagy, antioxidants serve as natural down-regulators of this process (Scherz-Shouval and Elazar, 2011). Autophagic could induced by oxidative stress in the common carp (*Cyprinus carpio* L.) (Chen et al., 2015). However, there is no evidence regarding the toxicity mechanism of autophagy activated by oxidative stress in *Carassius auratus* lymphocytes induced by PFOA.

The mechanism of intracellular autophagy can be divided into several steps, which include initiation, autophagosome formation, autophagosome–lysosome fusion, and degradation and recycling of cargo. Recycling is up-regulated when cells are during oxidative stress (Deretic et al., 2013). Approximately 30 proteins are involved in the regulation of this process. Once autophagy is initiated, phagophores lead to the formation of double-membrane vesicles referred to as autophagosomes, and Atg 5 and Atg 7 are crucial to the elongation of pre-autophagosomal structure (Klionsky et al., 2012). The second conjugation system leads to formation of the microtubule associated-protein 1 light chain 3 (LC3)-phosphatidylethanolamine conjugate. LC3-I is converted to LC3-II (autophagosome) through lipidation that involves Atg 7, allowing the association of LC3 with autophagy vesicles (Klionsky et al., 2012; Noda and Inagaki, 2015). Beclin 1, also known as BECN1, is a homolog of yeast Atg6 and is a specific gene for mammalian involvement in autophagy (Mizushima et al., 2010). It has been shown that the up-regulation of Beclin1 expression in mammalian cells can stimulate autophagy (Liang et al., 1999). Our results demonstrated the dose-dependent accumulation of Atg5, Atg7, and Beclin 1 at two concentration stages following exposure to PFOA, indicating the induction of the autophagic cascade following exposure to PFOA. When the exposure concentration ranges from 1 to 10 μg/L, the mRNA relative expression levels of Atg 5, Atg 7, and Beclin 1 were generally up-regulated (compared with the control group). The autophagy mechanism was induced by PFOA exposure, while the generation of intracellular ROS was increased slowly. However, when the exposure concentration reached 100 μg/L, the autophagy mechanism of lymphocytes was disrupted. It is speculated that autophagosomes cannot be catabolized by inhibiting the combination of autophagy and lysosomes after their formation, and the ROS content was increased significantly in the same time. Yan et al. (2015) reported that PFOA blocks autophagy and disturbs intracellular vesicle fusion in mouse liver at high cytotoxic concentrations (200 μM); this result cannot be detected after human exposure to the same dose and showed that *C. auratus* lymphocytes were more sensitive to PFOA than rat liver and that the human body has lower sensitivity.

In this study, we found that autophagy can be activated by oxidative stress induced by low doses of PFOA (1 and 10 μg/L) because the mRNA expression of Atg 5, Atg 7, and Beclin 1 was significantly up-regulated compared with that of the control. However, dysfunction under higher doses of PFOA exposure (100 μg/L) because the relative expression of autophagic genes (Atg 7 and Beclin1) presented a trend of down-regulation compared to low concentration treatment group. Scherz provides the first indication for the involvement of ROS in starvation-induced autophagy as signaling molecules in a survival pathway in starved CHO and HeLa cells because the increase in ROS was both local and reversible (Scherz-Shouval et al., 2007).

Partial correlation analysis was performed on eight parameters. The data were standardized using the minimum–maximum approach, and repetitions of each indicator were higher than or equal to six times. The results indicated the relevance of multiple variables. The ROS level was significantly correlated with the GSH content, SOD activity, and relative expression of Atg 5 and was significantly correlated with Atg 7. The cell growth inhibition rate was significantly correlated with another eight parameters. There was a significant internal correlation among the cytotoxicology, oxidative stress, and autophagy induced by PFOA in the lymphocytes of *C. auratus.* Additionally, the intracellular ROS and MDA levels were positively correlated with cytotoxicity and negatively correlated with the levels of antioxidant enzymes GSH and SOD. Oxidative stress was positively correlated with cytotoxicity in lymphocytes after exposure to PFOA. The relative mRNA expression levels of Atg 5, Atg 7, and Beclin 1 were negatively correlated with cytotoxicity, the intracellular ROS levels and the MDA levels, while they were positively correlated with the GSH and SOD levels. Autophagy was negatively correlated with cytotoxicity and oxidative stress in lymphocytes after exposure to PFOA.

In addition, the analysis showed that the Atg 5 sequence possessed an autophagy protein 5 domain (George et al., 2000). The Atg 7 sequence possesses an N-terminal domain of the ubiquitin-like modifier-activating enzyme Atg 7 that binds the E2 enzymes Atg10 and Atg3 in *Arabidopsis* (Ozeki et al., 2016). The beclin 1 sequence possesses a Beclin 1 BH3 domain, which is a short motif known to bind to Bcl-xLs. This interaction is important in apoptosis (Zalckvar et al., 2009). Additionally, the Beclin1 sequence has an autophagy protein Apg6 domain. Our results showed that Atg 5, Atg 7, and Beclin 1 mRNA expression significantly increased in the *C. auratus* lymphocytes exposed to PFOA. PFOA affects the autophagy systems of *C. auratus* lymphocytes by inducing the expression of autophagy signaling pathway genes mRNA levels.

**FIGURE 9:**
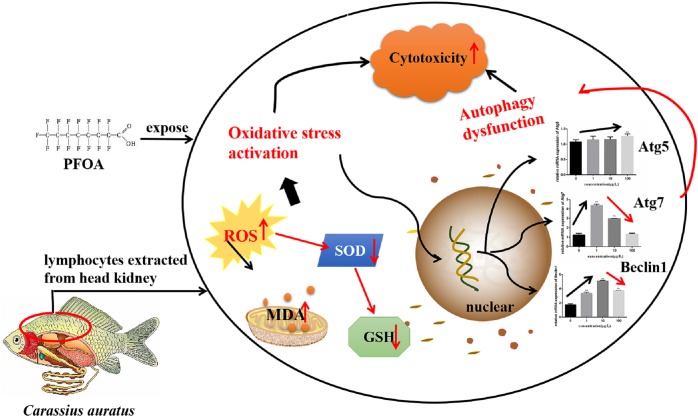
The proposed signaling pathway of regulating oxidative damage related to gene expression associated with the PFOA-induced autophagy signaling pathway in *C. auratus* lymphocytes.

In summary, current study demonstrated that PFOA causes obvious oxidative damage and caused the expression of Atg 5, Atg 7, and Beclin 1 imbalance to *C. auratus* lymphocytes. Autophagy signaling pathway-associated genes imbalance-related oxidative stress may be involved in the mechanism of *C. auratus* lymphocytes injury caused by PFOA (Figure 9).

## Author Contributions

JT designed the study, writing, and interpretation of the results. XL, FC, XY, DZ, JY, JH, BC, XS, JJ, and WL carried out most of the experimental work. HZ had overall responsibility for the project and editing the whole manuscript.

## Conflict of Interest Statement

The authors declare that the research was conducted in the absence of any commercial or financial relationships that could be construed as a potential conflict of interest.

## Footnotes

1 http://www.ncbi.nlm.nih.gov/gorf/gorf.html

2 https://www.ncbi.nlm.nih.gov/

3 http://www.clustal.org/clustal2/
